# Interprofessional team-based learning in basic sciences: students' attitude and perception of communication and teamwork

**DOI:** 10.5116/ijme.5f5b.24e3

**Published:** 2020-09-29

**Authors:** Lukas Lochner, Heike Wieser, Gabi Oberhöller, Dietmar Ausserhofer

**Affiliations:** 1 Claudiana, College of Healthcare Professions, Teaching Support Office, Bolzano/Bozen, Italy; 2 Claudiana, College of Healthcare Professions, Research Unit, Bolzano/Bozen, Italy; 3 Laimburg Research Centre, Science Support Centre, Vadena/Pfatten (BZ), Italy

**Keywords:** Medical education, basic sciences, anatomy education, interprofessional learning, team-based learning

## Abstract

**Objectives:**

To explore whether a team-based learning strategy applied to an interprofessional course on basic science changes students' perception of communication and teamwork skills and attitudes as related to interprofessional learning.

**Methods:**

A mixed-methods approach was utilized. The participants were selected through an opportunity sample of 33 first-semester anatomy students from occupational therapy and orthoptics programs. Students completed an interprofessional questionnaire before and after the course. The data were analyzed descriptively. Fourteen students were selected randomly for group interviews. Qualitative data was interpreted using thematic analyses.

**Results:**

The pre-test scores for 'communication and teamwork skills' and 'interprofessional learning' were high with mean values of 26.58 and 34.24, respectively. The post-test scores were 27.30 and 34.27, respectively, indicating no relevant changes in students' perception and attitudes. Qualitative data suggested that team-based learning represents a valid strategy to encourage communication and teamwork skills but revealed a lack of interprofessional exchange during the course. Students reported that classroom activities must require the professional knowledge of all participating groups in order to prevent a negative attitudinal shift towards interprofessional education in the later years of their studies.

**Conclusions:**

Implementing team-based learning in basic sciences can encourage communication and teamwork among students. Mixed classes can help socialize students of different professional groups, although they carry a risk of a negative attitudinal shift towards interprofessional education. Whether, and in what ways, effective interprofessional exchange during the teaching of basic sciences can be achieved needs further investigation.

## Introduction

In today's health care system, delivering high-quality patient care is the shared responsibility of various health care professionals, who are, consequently, expected to collaborate effectively.^1^ Effective interprofessional collaboration is assumed to have an impact on improving the quality of care and reducing the per capita cost of health care.^2^ Consequently, universities are increasingly committed to ensuring students graduate with the required collaboration skills, such as communication and teamwork.^3^ Interprofessional education (IPE) is defined as occasions when two or more professions learn with, from and about each other to improve collaboration and the quality of care.^4^ It has been advocated as a key means by which such collaboration skills can be fostered prior to entering the workplace.^5^^-^^7^ Commonly, attention has mostly focused on IPE in the later stages of the educational program.^1^ However, there are benefits to be gained from the introduction of IPE in the early years, as the literature indicates that students' readiness for IPE is high at the beginning of training but declines significantly over time.^8^^,^^9^ Consequently, there has been a call for integrating interprofessional learning objectives, such as communication and teamwork, into the basic sciences.^10^

Among the basic sciences, anatomy is key to many health care professions, and the literature suggests that the shared learning of anatomy can provide an excellent content vehicle to implement IPE in the early years of training.^11^^-^^15^ In order to enable students to understand the contribution that effective collaboration makes to problem-solving, their attention needs to be drawn not only to the content but also to the process of learning. Students should interact with each other in a way that fosters shared decision-making and listening to other team members.^5^^,^^6^ An active learning approach in which student activities are introduced into the classroom, in contrast to the traditional lecture where students passively receive information from the instructor, has the potential to improve communication skills, particularly when students are required to work cooperatively in small groups toward a common goal.^16^^,^^17^

An instructional method that facilitates active learning is team-based learning (TBL).^18^^,^^19^ TBL combines independent out-of-class preparation with in-class discussion in small groups and allows students to learn about working within teams. Since TBL also draws the attention of participants to the process of learning, it has been credited with an improvement in communication and teamwork skills.^20^^,^^21^ It also represents an attractive method for basic sciences because it promotes both learning of facts as well as the development of concepts for problem-solving, and medical schools have successfully adopted a TBL strategy in the delivery of anatomy.^22^^,^^23^

Recognizing the need for competency in interprofessional collaboration, in the academic year 2017-2018, students from two different health care professional programs at Claudiana - College of Healthcare Professions in Bolzano/Bozen, Italy, were brought together for a novel interprofessional course in anatomy implementing a TBL strategy. The first author (LL) was scheduled to teach the basic anatomy of organ systems in the occupational therapy as well as in the orthoptics program. This was seen as an opportunity to unite the students of both programs for this course, and to introduce 'communication and teamwork' as an interprofessional learning objective in addition to the learning goals of anatomy.

### Educational setting and course description

The College of Healthcare Professions in Bolzano/Bozen, Italy, offers three-year bachelor's programs in non-medical health professions. In the discipline-based curricula, the predominant teaching method during theoretical instruction is mandatory attendance in didactic lectures. The programs are strictly segregated.

This study refers to the basic anatomy course of human organ systems for students from the programs in occupational therapy and orthoptics. The learning goals for anatomy in both courses were identical (no dissection or other practical sessions were programmed, and after the course, students pursued their course-specific studies in anatomy). Compared to previous years, the content related to anatomy remained unchanged, while the development of communication and teamwork skills was added and explicitly stated in the course syllabus. To promote the interprofessional learning objective, we chose TBL as an educational framework. As we slightly modified the classical TBL to fit the local curricular needs and approach, the following section describes how the TBL sessions were constructed and integrated into the anatomy course. The description is based on the guidelines for reporting TBL activities.^24^

The anatomy course was organized into seven sequential organ system-based modules ('cardiovascular system', 'lymphatic system', 'respiratory system', 'digestive system', 'urinary system', 'reproductive system', and 'endocrine system'). The overall student workload was 50 hours (two credit points). Twenty-five hours were designated to self-study, and 25 hours were scheduled as in-class sessions (TBL). For each of the seven modules, online learning activities were created, which were available through a web-based learning system. The online learning was conceptualized as preparatory assignments to the in-class sessions and consisted of two sequential steps: (1) One self-made 10-minute online human anatomy video related to the respective organ system,^25^ and (2) One fill-in-the-blank assignment based on pictures of human anatomy. Assignments were created offline from the existing course material and then uploaded as PDF files. Students were requested to download the material and complete the exercise using a recommended anatomy textbook.

For the in-class TBL sessions, we stratified students according to their professional group and the results of an anatomy test at entry. We allocated them to one of the six teams, each containing five or six members. This way, each team consisted of three to four students from the occupational therapy program and two or three students from the orthoptics program, with prior anatomy knowledge evenly distributed between teams. Each team appointed a team spokesperson. All students were new to TBL. The in-class TBL sessions consisted of three distinct parts.

Each session began with the readiness assurance process. The Individual Readiness Assurance Test (IRAT) entailed two fill-in-the-blanks exercises based on pictures of human anatomy that focused on the factual contents from the online preparatory assignments. Each exercise displayed an anatomy image with 2 to 4 structures to identify. After this, students completed the Group Readiness Assurance Test (GRAT) by taking the same two exercises as a team. Following the GRAT, the instructor asked the team spokesperson to relay their team's answers verbally. If all team's answers were correct, then the instructor either moved to the next question or asked a supplementary question. If any team's answers were incorrect, he would give immediate feedback rectifying any errors. Students then filled out a scoring sheet, in which a maximum of two points were awarded for each exercise done correctly. The sheet contained the results for the IRAT of each student as well as the result of the GRAT.

Thus, the students could compare the individual scores with the team score (the teams almost always reached the maximum score, while the individual students usually scored lower).

Following the readiness assurance process, practical demonstrations with medical mannequins and human organ models reinforced the knowledge of the anatomy of the respective organ system. This constituted the second part of the in-class session. At this juncture, the focus was on the understanding and application of anatomical knowledge by putting it into a pathophysiological or clinical context.

The third part of the in-class session was dedicated to a written application exercise. Here, the teams were confronted with a pathophysiological or clinical query on paper that related to the day's topic. The query was crafted to require integration of anatomical facts and concepts. Teams were asked to select the most appropriate answer from a given list (multiple choice), and each team worked on the same problem to make a specific choice. The team solutions were reported simultaneously to the whole class by holding up colored letter-cards. If all teams displayed the same answer, the instructor asked further questions to stimulate discussion. If teams disagreed, the discrepancies were addressed by asking the teams to defend their answers, followed by discussion and feedback.

As this was a pilot project, the IRAT and the GRAT were not graded and students were not offered the possibility of appeal (however, team scores of the application exercise were calculated at the end of the course, and the "winning" team received a small traditional Italian Christmas cake as a reward). In addition, we did not include any peer review element in the project, as this has the potential to create tensions among students, especially when they are not used to evaluating each other's work.^23^^,^^26^ We deemed it sufficient that the GRAT and the application exercise required students to communicate effectively and work with other team members. That is, communication and teamwork exercises were not graded. As in previous years (i.e., uniprofessional anatomy courses using the same flipped classroom and blended learning strategies, but without TBL-activities integrated) the assessment of anatomy learning goals consisted of a combination of written and oral end-of-term exams. Although we designed this study to explore interprofessional learning objectives only, we identified a comparable historical cohort of students from the occupational therapy program (academic year 2014-2015) and compared the results of the written end-of-term exam with the results of the occupational therapy students from this study. The historic cohort scored an average of 87.56 points out of 100 (n=19), while the students from this study scored 87.45 points (n=19). In conclusion, the comparison of this historical cohort to other previous courses (from other professional groups) did not yield any significant difference in student anatomy grades.

### Rationale and study aims

The general objective of this study was to clarify if and how this course could contribute to the interprofessional learning outcomes of the programs as it was not clear how our students perceive and value the IPE-experience in these early stages of training. We were interested whether we were able to foster communication and teamwork skills among first-year students during the education of basics sciences thereby justifying the organizational effort to unite students from different programs for common courses in the future.

Few studies have assessed the use of anatomy as a means of IPE.^11^^-^^15^ To our knowledge, no previous study has applied interprofessional TBL as an instructional framework for anatomy education. This article describes the implementation of interprofessional TBL in a single basic anatomy course. In particular, the aims of our investigation were three-fold: (1) Do students' self-assessment of communication and teamwork skills change?, (2) Do students' attitudes toward interprofessional learning change?, and (3) How do the perspectives of the students help explain possible changes?

## Methods

### Study design and participants

To generate a comprehensive picture of the student' perspectives, we utilized a mixed-methods design combining a quantitative with a qualitative approach.^27^ We distributed a questionnaire before and after the anatomy course to explore possible changes in students' self-assessment regarding ‚communication and teamwork' and changes in their attitude towards ‚interprofessional learning'. The content and results from the survey led to the construction of an interview guide. In an effort to understand in more depth the possible changes in the students' perceptions, we followed up with group interviews to elicit a variety of opinions in an interactive setting.^28^

The study protocol was approved by the Institutional Supervisory Board of Claudiana – College of Healthcare Professions (protocol 9/1/2017). Students who participated in the study provided written informed consent. To guarantee their anonymity no personal data (e.g., names, e-mail addresses or phone numbers) were collected. Pseudonyms were assigned for interviews, and all identifying information was omitted in the transcripts. The course was delivered as part of the regular curriculum. Learning objectives, content and contact time in the classroom were not changed due to the study.

The first author (LL) was scheduled to teach basic anatomy of organ systems in the occupational therapy as well as the orthoptics program. A total of 33 first-semester students from both of the three-year bachelor's programs were combined into one course, thus creating an opportunity sample. Twenty students were from the occupational therapy program, and 13 students were from the orthoptics program. All 33 students filled out the pre-test questionnaire (response rate 100%), 30 students filled out the post-test questionnaire (17 occupational therapy students and 13 orthoptics students), with a response rate of 91%.

After the completion of the course, eight occupational therapy students and six orthoptics students were randomly selected and asked to take part in the group interviews. All of them agreed to participate. We decided to interview students from the different programs separately in order to encourage an uninhibited discussion in a group setting with familiar colleagues from their own study program. This led to the creation of two groups (i.e., one interview group of eight occupational therapy students and one interview group of six orthoptics students).

### Data collection and analysis of the survey

We asked students to fill out structured questionnaires on paper one week before the course (pre-test) and three days after it (post-test). Variables and measures were taken from the German version of the 'University of the West of England's Interprofessional Questionnaire (UWE-IP)'.^29^^,^^30^ This instrument is designed to measure students' attitudes to interprofessional education.^31^ It consists of four subscales: Communication and Teamwork, Interprofessional Learning, Interprofessional Interactions, and Interprofessional Relationships. For this study, we only applied the first two subscales (i.e., Communication and Teamwork, Interprofessional Learning) as the first-semester students did not have enough contact with other health professionals to enable them to assess meaningfully the other two subscales.^32^ Pollard and colleagues reported Cronbach's alpha coefficients equal to 0.76 and 0.84 respectively for the two subscales used.^29^ The Communication and Teamwork Scale consists of nine items, using a four-point Likert scale where statements are scored from 1 (strongly agree) to 4 (strongly disagree). Since it is assumed that all respondents will have experience of communication and group work at an informal level, the neutral point is omitted. The minimum score for this scale is 9, while the maximum is 36. Scores from 9 to 20, from 21 to 25, and from 26 to 36 are considered to indicate respectively negative, neutral and positive self-assessment of communication and teamwork skills. The Interprofessional Learning Scale also consists of nine items but uses a five-point Likert scale where statements are scored from 1 (strongly agree) to 5 (strongly disagree), the neutral point is included. The minimum score for this scale is 9, while the maximum is 45. For this scale, scores from 9 to 22, from 23 to 31, and from 32 to 45 indicate respectively negative, neutral and positive attitudes towards interprofessional learning.

We collected data from the questionnaires for analysis. All reverse-coded items were recoded. We calculated mean scores for all single items of both scales and overall scores for both scales and studied the results of the pre- and the post-tests. As we observed very similar values in pre- and post-results for all single items as well as for the overall scores, we decided not to apply further statistical elaboration and to opt for descriptive analyses (i.e., mean, standard deviation) only.

### Data collection and analysis of the interviews

The two group interviews were moderated by three of the authors (HW, GO, DA), using an interview guide that was based on the content and results of the survey. None of these individuals was involved in the organization or teaching of the respective anatomy course. Prior to the interviews, the first author (LL) familiarized the moderators with the TBL features of the course. The interviewers began the group interviews with an open-ended question: "What was your experience of this anatomy course?", which was aimed at examining the participants' perceptions of the course in general. Further questions focused on communication and teamwork during in-class sessions as well as studying outside class, and aspects relating to interprofessional learning during the course and outside the course. For example, questions included: "Did you enjoy working in teams?", "Who led the groups, and how were decisions taken?", "Did you learn anything from or about the other professional group?", and "Did you meet or communicate with students from the other professional group outside the class?". When deemed necessary, the interviewers encouraged the participants to elaborate on their thoughts and perspectives in more detail. The first group interview with the occupational students lasted 50 minutes. Following this initial experience, we reviewed the interview guide and slightly adjusted it. The second group interview with the orthoptics students lasted 35 minutes. Both interviews were audio-recorded and transcribed verbatim.

The analytic procedure was based on the thematic analysis approach as described by Braun and Clarke.^33^ As the group interviews were intended to help explain the quantitative results, we included two predetermined topics from the questionnaire in the qualitative analysis (i.e., 'Communication and Teamwork', and 'Interprofessional Learning').^27^ Steps in the qualitative analysis comprised: (1) the transcripts were read repeatedly by the first author (LL) to facilitate a clear understanding of the data; (2) he coded the data and allocated the codes to the predetermined topics; (3) he searched for themes within the predetermined topics; (4) the definition and context of codes and themes were established, and then discussed and reviewed with the other authors who were present during the interviews (HW, GO, DA); (5) all authors agreed on a definition of a final thematic outline that was used as a basis for a narrative reproduction of the results. In order to member-check the analysis, we presented a written one-page summary of the qualitative results to all participants of the interviews (except one orthoptics student who dropped out of college shortly after the conclusion of the first year). All students fully agreed with our analysis.

## Results

### Quantitative results

Communication and teamwork - To explore students' self-assessment of 'communication and teamwork' and possible changes in the pre- and post-test, we calculated and analyzed the mean scores of this scale. The pre-test demonstrated a value of 26.58, indicating a positive self-assessment of communication and teamwork skills. The post-test score was 27.3, indicating no relevant change. Table 1 displays the mean values and standard deviations for each single item.

**Table 1:** Pre- and post-results of the communication and teamwork subscale of the UWE-IP questionnaire

Communication and teamwork	Pre (N=33) Mean (SD)	Post (N=30) Mean (SD)
I feel comfortable justifying recommendations/advice face to face with more senior people	2.36 (0.74)	2.80 (0.76)
I feel comfortable explaining an issue to people who are unfamiliar with the topic	3.33 (0.59)	3.30 (0.60)
I have difficulty in adapting my communication style (oral and written) to particular situations and audiences (R)	3.21^*^ (0.54)	3.13^*^ (0.73)
I prefer to stay quiet when other people in a group express opinion that I don't agree with (R)	3.03^*^ (0.64)	3.03^*^ (0.56)
I feel comfortable working in a group	3.36 (0.49)	3.30 (0.60)
I feel uncomfortable putting forward my personal opinions in a group (R)	3.12^*^ (0.60)	3.30^*^ (0.53)
I feel uncomfortable taking the lead in a group (R)	2.24^*^ (0.80)	2.50^*^ (0.73)
I am able to become quickly involved in new teams and groups	3.09 (0.58)	3.10 (0.48)
I am comfortable expressing my own opinions in a group, even when I know that other people don't agree with them	2.82 (0.73)	2.83 (0.60)
Total	26.58	27.30

Note:^ *^values were recoded; R: item is reversed

Interprofessional learning - To explore students' possible changes in attitude in relation to 'interprofessional learning' in the pre- and post-test, we also calculated and analyzed mean scores of this scale. The pre-test results demonstrated a value of 34.24, indicating a positive attitude with regard to interprofessional learning. The post-test score was at 34.27, indicating no relevant change in attitude after the anatomy course. Table 2 displays the mean values and standard deviations for each single item.

### Qualitative results

The analysis of the interviews revealed four major themes. We allocated to each predetermined topic, i.e., 'Communication and Teamwork' and 'Interprofessional Learning', two themes. The following description is a summary of what the participants expressed during the two group interviews (GI).

**Table 2:** Pre- and post-results of the interprofessional learning subscale of the UWE-IP questionnaire

Interprofessional learning	Pre (N=33) Mean (SD)	Post (N=30) Mean (SD)
My skills in communicating with patients/clients would be improved through learning with students from other health and social care professions	3.24 (0.94)	3.33 (0.99)
My skills in communicating with other health and social care professionals would be improved through learning with students from other health and social care professions	4.09 (0.58)	3.93 (0.91)
I would prefer to learn only with peers from my own profession. (R)	3.45* (1.15)	3.70* (0.95)
Learning with students from other health and social care professions is likely to facilitate subsequent working professional relationships	4.15 (0.71)	4.07 (0.64)
Learning with students from other health and social care professions would be more beneficial to improving my teamwork skills than learning only with my peers	3.33 (0.99)	3.30 (0.80)
Collaborative learning would be a positive learning experience for all health and social care students	4.09 (0.80)	4.0 (0.83)
Learning with students from other health and social care professions is likely to help to overcome stereotypes that are held about the different professions	4.12 (0.78)	4.13 (0.63)
I would enjoy the opportunity to learn with students from other health and social care professions	3.85 (0.75)	3.87 (0.90)
Learning with students from other health and social care professions is likely to improve the service for patient/client	3.91 (0.80)	3.93 (0.78)
Total	34.24	34.27

Note: *values were recoded; R: item is reversed

### Communication and teamwork

#### (1) Group learning versus individual learning

The first theme related to the learning process during group assignments in class. Most participants felt that learning was fostered by group work. This was because "things are elaborated upon at the time, and the thinking process is immediately apparent" (GI 1, occupational therapy students). Students also stated that they benefitted from knowledgeable colleagues because when contents remained unclear after the teaching part of the in-class sessions they "discussed these issues in the group, which was very helpful" (GI 2, orthoptics students). One student stated that the group discussion "was a pleasant experience, very different from the usual didactic lectures" (GI 1). Participants emphasized that these discussions were particularly stimulated by the third part of the in-class sessions, i.e., the application exercise, in which the teams were requested to reach a collective decision. Although most participants reported that they appreciated working with other people in a team, a few students held the opinion that this might depend on one's personality. Two students even reported that they are "people who prefer to work individually" (GI 2). Nonetheless, participants were aware that teamwork would be of importance for them in their future career. One student, for example, stated that "working in a group will be useful later on in the working world" (GI 2). In any case, the group learning was limited to the anatomy in-class sessions as none of the participants reported having any meetings with the group outside class.

#### (2) Formal group leader versus opinion leader

The second theme related to the process of group leading. Teams appointed a formal team spokesman who moderated the group discussions, marked the score sheet and presented the team's answers. However, participants reported that usually, the students with the most anatomical background knowledge became opinion leaders, especially during the application exercise. Participants stated that the other group members usually "thought for themselves and expressed their opinion during group discussions", but eventually "trusted" the students with the most prior anatomical knowledge as they were able "to offer plausible reasoning" (GI 1). However, not all participants agreed that everyone in the team freely expressed their own view, as one student, for example, stated "you sometimes remain silent and agree with the group decision despite having a different opinion" (GI 2). When teams were unable to reach a consensus, they voted to arrive at a group answer to present.

### Interprofessional learning

#### (1) Interprofessional learning versus uniprofessional learning

The first theme related to learning from and about the other professional group. Most students reported that "it was nice to work with new people" (GI 1) but being together in class with students from a different profession was not relevant to learning the content of the course. They "went to class to learn anatomy" (GI 2) and felt that both professional groups "had about the same level of anatomical knowledge" (GI 1). One participant stated that she might be "less anxious to approach students from the other professional group if she needed to" (GI 1), but in general students did not perceive it "necessary to sit in class with another professional group" (GI 2). However, during the interviews, it became evident that a kind of informal interprofessional exchange did occur during group work. One student reported that when the team members introduced themselves, they "told each other why they had chosen to study this profession" (GI 1). Another participant stated that they "had talked about their studies and how they were structured" (GI 2). None of the participants reported that they met students from the other professional group outside class, as they did not perceive this necessary to achieve the anatomy learning objectives. In addition, they reported that tight schedules and spatial separation (the two educational programs are hosted in different buildings) impeded contact between the professional groups.

#### (2) Implementation of interprofessional education

The second theme related to the participants suggestions for measures to support the implementation of interprofessional initiatives, in which students not only learn with but also from and about each other. In general, participants emphasized that they believed that working with other health care professionals will play an important role in their future career. Several students stated that this collaboration needs to be realized professionally, and that "you don't need to be best friends" (GI 2) in order to achieve this. Participants stated that the professions selected to learn together should have as many "areas of overlap" (GI 1) as possible. One participant, for example, proposed choosing students from professions that "work together in rehabilitation later on" (GI 1). In order to understand in "what ways the professions work together" (GI 2) several students stated that they needed to be presented at the beginning of the course with "real-life examples through which one can see how each profession is helpful" (GI 2). Many participants believed that interprofessional courses would make more sense later on in their studies, when they have "a better idea about their own professional profile" (GI 2) and can "tell the other profession how their own profession can contribute "(GI 2). Suggestions to foster interprofessional exchange at the beginning of the educational program included organizing "a one-day-visit" (GI 2) to the other professional group as well as scheduled "class-room presentations during which each group outlines its professional profile" (GI 1) to students from another profession. Many students also believed that it would be very helpful if students that were selected to learn together were also located spatially close together (i.e., in the same building), with schedules that allow for enough spare time to meet informally outside class.

## Discussion

Interprofessional team-based learning was applied in a basic science course of anatomy with students from two health professions with the goal of promoting interprofessional learning outcomes related to 'communication and teamwork' and to enhance students' attitudes to interprofessional education.

The quantitative findings of this study indicated a positive self-assessment of communication and teamwork skills before the course. There was no indication that the course altered this value; however, qualitative data suggested that TBL represents a valid strategy to encourage team and communication skills. The results are in line with findings of previous studies that found that implementing a TBL strategy encourages students to keep up with the course assignments, provides an opportunity to apply knowledge, and thus creates an environment of increased engagement.^34^^,^^35^ The results suggest that 'communication and teamwork' can be fostered

in the early years of training, during the teaching of the basic sciences, and that TBL can be regarded as a valuable alternative to didactic lectures in anatomy education with the advantage of further developing these interprofessional learning objectives.

Among the basic sciences, anatomy is regarded as a convenient common content vehicle for interprofessional education as all health professional students require a basic understanding of the structure of the human body.^13^ However, the quantitative results of this study indicated that the course did not lead to positive attitudinal changes in relation to 'interprofessional learning'. The qualitative data from the interviews revealed a plausible explanation for this finding: some students did not find it necessary to sit in class with students from another program in order to learn anatomy. The literature suggests that balanced exchanges between the involved professions are important in order for the students to experience learning from the other group and, consequently, appreciate the value of interprofessional settings.^1^^,^^30^ Qualitative data from this study confirmed this proposition for the context of this course. The need for pathophysiological or clinical cases that require knowledge from both participating professions was repeatedly noted by interview participants. Ideally, such cases ensure that the professional knowledge of all participating groups is necessary, thus making learning from each other central to completing the activity.^32^^,^^36^ Otherwise peer teaching takes place only between students with more previous content knowledge (in this case prior anatomy knowledge) and students with less content knowledge, without necessarily triggering a constructive dialogue between the different professional groups involved.^14^ In this course, participating students reported that TBL promoted communication and teamwork, but this effect was rather confined to intra-professional groups and did not lead to specific interactions with the students from the other professions. Perhaps the "distance" between the professional domains, i.e., occupational therapy and orthoptics, was too great; it might be more fruitful to involve students from professions that are more closely related to each other, e.g. occupational therapy and physical therapy.

However, the interview data also suggested that many students appreciated having some instruction outside their usual classroom setting, and, in this way, getting to know students from a different program. A few interview participants stated that some informal exchange between students concerning the different study programs occurred. Informal communication can nurture positive group dynamics.^9^ It seems reasonable to teach some basic sciences, like anatomy, in mixed classes to enhance socialization and help prevent the formation of prejudices. However, in order to encourage first-year health professions students to learn from and about the other professional group, as required for genuine interprofessional education, a basic science by itself might not provide adequate means. In this study, the quantitative results indicated that students' attitude to interprofessional learning did not worsen, but from qualitative data it became apparent that there is a risk that students in mixed classes perceive IPE as disadvantageous when their own professional learning is impeded. This finding is in line with literature suggesting that in mixed classes students might perceive that their learning opportunities are being diluted.^1^^,^^37^ Hence, when creating IPE courses for basic sciences, what content to include for which students must be critically evaluated in order to create a positive culture of exchange and increase students' readiness to engage with IPE in the later years of their studies.

### Limitations

This descriptive article reports on a single pilot educational experience at a single institution, making it impossible to generalize the results to other educational venues. An important limitation of this report is the small number of learners in the class, which only allowed descriptive statistics. Furthermore, only the learners' perceptions were assessed. Studies of higher-order constructs (such as learning, behavior and results) will be necessary to measure more accurately the effectiveness of the TBL strategy in fostering interprofessional skills related to communication and teamwork in courses of basic sciences.

## Conclusions

This study revealed that basic sciences, such as anatomy, can serve as a content vehicle to implement team-based learning, with the potential to foster skills in communication and teamwork among students. Interprofessional classes in the early stages of training can enhance students' socialization between professional groups, justifying the organizational effort to unite students from different programs. In mixed classes, however, great care must be taken to prevent the perception by students that their own professional learning is impeded, as this might result in a negative attitudinal shift towards interprofessional education for the later years of their studies. The results also revealed that the subjects of basic sciences might be located too early in the curriculum in order to achieve genuine interprofessional learning, i.e. learning from and about each other, as this requires classroom activities where professional knowledge of all participating groups are necessary to complete the assignments. Further studies are needed to investigate whether and how students' learning from and about other professional groups during the teaching of basic sciences can be achieved.

### Acknowledgements

The authors thank Katherine Pollard, Senior Research Fellow at the Faculty of Health and the Applied Sciences University of the West of England, for the permission to use the University of the West of England Interprofessional Questionnaire (UWE-IP), and the Department of General Practice and Health Services Research, University Hospital Heidelberg, Heidelberg, Germany for providing us with permission to use the German version (UWE-IP-D). The authors also thank the first-semester occupational therapy and orthoptics students for their eager participation in this interprofessional learning experience.

### Conflict of Interest

The authors declare that they have no conflict of interest.
